# Lord Knutsford's 'Impossible'

**Published:** 1920-01-10

**Authors:** 


### THE EDITOR'S LETTER-BOX.

[Correspondence on all subjects is invited, but we cannot in any way be responsible for the opinions expressed by our correspondents, who must give their name and address as a guarantee of good faith, but not necessarily for publication. Correspondents are reminded that brevity of style and conciseness of statement greatly facilitate early insertion.]

### "LORD KNUTSFORD'S 'IMPOSSIBLE.'"

To Ierne :—You tell me that a Forty-Eight Hours Bill has passed in California for nurses. Of course hospitals at very great expense in salaries, housing, and feeding could be compelled to arrange for a forty-eight hours week—[what about Sundays, or are patients not to be nursed on Sundays?]. But will "Ierne" tell us whether the same law applies to private nursing, and whether every patient therefore is compelled to engage three nurses where two did the work before. As I have ventured to point out to "Ierne" before, I have had a very large personal knowledge of nurses at my house. I have had no less than thirty of them to nurse my family and myself, and I have never found the twelve-hours' shift either too trying or exhausting. The day nurses, of course, had time off during the twelve hours. "What would he have cared if they had been tired or exhausted?" may be asked. And I will not answer, because those who do not know me, or who believe in Mrs. Bedford Fenwick's opinion of me, would not credit me whatever I replied—and because those who do know me will not require an answer.

Knutsford.

